# *In Utero* Exposure to Diethylhexyl Phthalate Affects Rat Brain Development: A Behavioral and Genomic Approach

**DOI:** 10.3390/ijerph121113696

**Published:** 2015-10-28

**Authors:** Han Lin, Kaiming Yuan, Linyan Li, Shiwen Liu, Senlin Li, Guoxin Hu, Qing-Quan Lian, Ren-Shan Ge

**Affiliations:** Department of Anesthesiology, The Second Affiliated Hospital & Yuying Children's Hospital of Wenzhou Medical University, Wenzhou 325000, Zhejiang, China; E-Mails: linhanwenzhou@wzhealth.com (H.L.); yuankaiming@aliyun.com (K.Y.); gerenshan2@163.com (L.L.); shiwenliu77@gmail.com (S.L.); lisenlinwenzhou@163.com (S.L.); hgx@wzmc.edu.cn (G.H.)

**Keywords:** DEHP, proliferation, cognitive function, rat, brain development

## Abstract

Diethylhexyl phthalate (DEHP) is one of the most widely utilized phthalate plasticizers. Previous studies have demonstrated that gestational or postnatal DEHP exposure induced adverse effects on rat brain development and function. In this study, we investigated the effects of gestational DEHP exposure on gene expression profiling in neonatal rat brain and cognitive function change at adulthood. Adult Sprague Dawley dams were orally treated with 10 or 750 mg/kg DEHP from gestational day 12 to 21. Some male pups were euthanized at postnatal day 1 for gene expression profiling, and the rest males were retained for water maze testing on postnatal day (PND) 56. DEHP showed dose-dependent impairment of learning and spatial memory from PND 56 to 63. Genome-wide microarray analysis showed that 10 and 750 mg/kg DEHP altered the gene expression in the neonatal rat brain. *Ccnd1* and *Cdc2*, two critical genes for neuron proliferation, were significantly down-regulated by DEHP. Interestingly, 750 mg/kg DEHP significantly increased *Pmch* level. Our study demonstrated the changed gene expression patterns after *in utero* DEHP exposure might partially contribute to the deficit of cognitive function at adulthood.

## 1. Introduction

Di-(2-ethylhexyl)-phthalate (DEHP) is one of the most widely utilized phthalate plasticizers of polyvinyl chloride plastic. DEHP is used in a wide range of consumer products, including medical catheters, infusion containers, and toys. Increasing concern has been expressed for its possible toxicity to multiple organs, including testes [1] and brain [2,3]. The fetal stage is a critical period, during which the fetus is vulnerable to the insult of environmental xenobiotics [4]. Many xenobiotics can elicit neurotoxicity through interfering with the cascade of brain developmental processes, including proliferation, migration, differentiation, and synaptogenesis [5]. Alteration in any of these processes may result in neurological dysfunction in later life [6,7].

Once DEHP is absorbed by mammals, it is readily metabolized to mono-(2-ethylhexyl)-phthalate (MEHP) by hydrolysis [8]. DEHP and its metabolite, MEHP, are able to freely cross the placental barrier of rats [9] and subsequently affect fetal development. This could happen in humans too, since similar DEHP was detected in the blood of pregnant women and neonatal cords [10]. DEHP levels were 1.15 ± 0.81 µg/mL in maternal plasma and 2.05 ± 1.47 µg/mL in the cord plasma [10]. 

The brain is one of the organs that may be susceptible to DEHP insult. Several studies in rodent models implicate that DEHP blocks brain development [2,11,12]. *In utero* exposure to DEHP (1500 mg/kg) was found to cause metabolic disturbance of lipid metabolome in the fetal brain [2]. Some of the lipids and fatty acids, including free cholesterol, sphingomyelin, and docosahexaenoic acid that are essential for brain development, were declined after DEHP exposure [2]. Postnatal exposure to DEHP also caused neurodegeneration in rats [12]. Epidemiological study showed that prenatal exposure to phthalates was negatively associated with altered behavior in boys [13]. However, whether prenatal exposure to DHEP can cause the deficit in cognitive function at adulthood and the possible mechanism is still unclear. The objective of the present study was to investigate the effects of in utero exposure to DEHP on the cognitive function changes at adulthood and possible alterations of gene expression levels in the neonatal brain.

## 2. Materials and Methods

### 2.1. Animals and Treatment

DEHP was purchased from Sigma-Aldrich (St. Louis, MO, USA). Adult Sprague Dawley dams were purchased from Beijing Vital River Laboratories (Beijing, China). All studies were approved by the Wenzhou Medical University Animal Care and Use Committee. Pregnant dams were divided into three groups of 14 rats: control (vehicle, corn oil), 10 and 750 mg/kg DEHP. The doses of DEHP were selected based on the fact that the lowest-observed-adverse-effect-level (LOAEL) of DEHP as a stimulatory effect on fetal testis, a sensitive organ, was 10 mg/kg, and the LOAEL as an inhibitory effect was 750 mg/kg [1]. DEHP was administered daily by gavage from gestational day (GD) 12 to 21 with 0 (control), 10 or 750 mg/kg DEHP in 1 mL/kg corn oil. The body weights of pups were measured on postnatal day (PND) 1 (at birth). One male pup per dam was euthanized by CO_2_ on PND 1, and its brain was removed and frozen immediately in liquid nitrogen for gene expression profiling and real time PCR (qPCR) analysis. 10 male pups per group were selected and retained for memory test in a Morris water maze on PND 56. 

### 2.2. Water Maze Testing

The Morris water maze [14] was utilized to evaluate learning and spatial memory abilities of rats at adulthood, with slightly modification from methods of Frye [15] and Li [16]. It consisted of a tank (diameter 110 cm–depth 50 cm) filled with water in a brightly lit testing room with several extra-maze distal cues (door, shelving, desk, and video-camera mounted on ceiling). The inner tank was equally divided into four quadrants. A black platform (12 cm–height 30 cm) was situated in the middle of quadrant I, 2 cm below the surface of water. A video-camera was setup above the water tank, and was connected to a computer tracking system (Shanghai Jiliang Software Technology Co., Ltd., Shanghai, China). The tracking program was used to measure the escape latency, path-length, swim-speed, and times across the platform as well as searching time in each quadrant. The video-camera also recorded the cues.

During the experiment, water in the tank was warmed up to 20–24 °C, and made opaque by adding black ink before tests so that the platform could not be seen. Ten male rats in each group mentioned above were used for water maze testing. On PND 56, rats were habituated to swimming in the pool for 120 s. Habituation consisted of a free swim, without platform in the tank. After the platform was re-fixed, rats were trained to find the hidden platform over four consecutive trials. During these training trials, rats had 120 s to search the hidden platform, starting from different quadrants on the edge of the pool. Rats that didn’t find the platform during training were guided to it by the experimenter. All rats remained on the platform for 15 s after each training trail. Then, two typical protocols, described as spatial reference memory and probe trials, were adopted for testing from PND 57 to PND 63. For the spatial reference memory test, four trials each day were performed in five consecutive days from PND 57. Rats were placed into water facing the wall of the tank, from different quadrants with 1h interval. In the meantime, the tracking program was initiated. Rats that could not find the platform within 120 s were guided to it. Once reaching the platform, the rats were allowed to remain there for 15 s. Performances of four trials each day for each rat were averaged for further analysis. For probe trials, the escape platform was removed from the tank. Rats were placed into water from the opposite quadrant (quadrant III) of the platform. Typically, a well-trained rat would swim to quadrant I and repeatedly cross the former location of the platform. This test was performed on PND 63. Rats were allowed to swim within 120 s. In these two tests, parameters were recorded automatically. After completing the trials, the rats were placed back into their cages.

### 2.3. Whole Genome Profiling Analysis

Total RNAs were extracted from rat brain by using TRIzol (Invitrogen, Carlsbad, CA, USA) according to the manufacturer’s protocol. Four samples from each group were randomly selected for a whole genome expression assay. RatRef-12 Expression BeadChip from Illumina Inc. (San Diego, CA, USA) was used. Biotin-labeled cRNA samples were prepared prior to hybridization. Briefly, 300 ng of each total RNA sample was used for cDNA synthesis, followed by 10 h of *in vitro* transcription to make biotin-labeled cRNA using an Illumina TotalPrep RNA Amplification Kit (Ambion Inc., Austin, TX, USA). The concentration of cRNA was measured using a NanoDrop2000 spectrophotometer (Thermo Fisher Scientific Inc., Wilmington, DE, USA). Amplified labeled cRNA (150 ng/µL) was then prepared in 10 µL GEX-HYB buffer (Illumina, Inc.) to a final concentration 30 ng/µL. Before being loaded to the BeadChip, the cRNA samples were preheated at 65°C for 5 min and cooled to room temperature. Then, the cRNAs were hybridized with the BeadChip for 18 h at 58 °C. After hybridization, the chip was washed in High Temp Wash buffer at 55°C, in 0.3% Wash E1BC buffer and in 100% ethanol at room temperature in turn. Followed by another wash in 0.3% Wash E1BC buffer, the chip was blocked for 10 min in 4 mL of Block E1 buffer. Array signals were developed by 10 min of incubation in 2 ml Block E1 buffer with 1 µg/mL Cy3-streptavidin (GE Healthcare, Piscataway, NJ, USA) solution. The chip was washed in 0.3% Wash E1BC buffer again, dried, and scanned. The Illumina BeadChip, comprising 12 microarrays on a glass slide, was scanned on an Illumina^®^ BeadArray™ Reader (Illumina, Inc.). Images were captured and the bead signals were analyzed using Illumina BeadStudio (San Diego, CA, USA). 

### 2.4. Microarray Analysis

Illumina microarray quality was determined by control bead analysis (housekeeping, hybridization, signal generation, and background). Arrays with overall intensity outliers from the majority of arrays (caused by poor hybridization conditions or poor imaging) were excluded from further analysis. Fourteen housekeeping genes were used to check the intactness of the biological sample. In brief, after background subtraction, the sample intensities were normalized using average method and resulting data were further analyzed with Illumina BeadStudio. Microarray quality was determined by data shown in the control table. Probes with detection p-value less than 0.05 were considered to be detectable.

### 2.5. Primer Selection and qPCR Verification

Some genes, including *Scyl1*, *Pmch*, *Cyp26b1*, *Tnp2*, *Cnih2*, *Neurod1*, *Cdc2*, *Ccnd1*, were randomly selected for validation by qPCR. All primers in this study were designed using the Primer 3 software (Whitehead Institute for Biomedical Research, Cambridge, MA, USA). The primers for *Scyl1* (forward: 5′-TCA TGG GGG TTA CAC CAG AT, reverse: 5′-CTT TGA CTG CTC TGC TGC TG), *Pmch* (forward: 5′-TCG GTT GTT GCT CCT TCT CT, reverse: 5′-TTC CCT CTT TTC CTG TGT GG), *Cyp26b1* (forward: 5′-GCC ATC AAT GTG TAT CAG GAG, reverse: 5′-GGT ACA CTG AAG CTT CTC ACG), *Tnp2* (forward: 5′-GGC CTC AAA GTC ACA CCA AT, reverse: 5′-TTC CCT TCC AAG GTC TTC CT), *Cnih2* (forward: 5′- CAT GAG CCC TTG GAG AGA AG, reverse: 5′-TCT TGG AGG AAT CTG GAT GG), *Neurod1* (forward: 5′-GGA TGA TCA AAA GCC CAA GA, reverse: 5′-CAG GGT ACC ACC TTT CTC A), *Cdc2* (forward: 5′-CTG GCC AGT TCA TGG ATT CT, reverse: 5′-CCG AAA TCT GCC AGT TTG AT), *Ccnd1* (forward: 5′-cgc gta ccc tga cac caa tct, reverse: 5′-cag aag cag ttc cat ttg ca), were used. Six total RNA samples in each group were randomly selected for qPCR assay. An aliquot of 1 µg total RNA in each sample was used for first strand cDNA synthesis and qPCR was performed as described previously [17]. The SYBR Green PCR Core Reagents kit purchased from Applied Biosystems (Foster City, CA, USA) was used with ribosomal protein S16 (*Rps16*) as an endogenous control. QPCR was carried out in a 25-µL volume using a 96-well plate. Primer titration was performed and the concentration of 300 nM was selected. *Rps16* mRNA levels were assayed in all samples as described previously [18]. Fluorescence was detected using an ABI 7700 system (PE Applied Biosystems, Carlsbad, CA, USA). Each sample was run in duplicate, in parallel with no template controls. Standard curves were performed for each run to ensure the comparability of the samples according to our previously published method [1]. All the individual results were evaluated against the standard curve. The expression levels were normalized to *Rps16*. The specificities of qPCR products were confirmed by both a single dissociation curve of the product and a single band with agarose gel electrophoresis. 

### 2.6. Statistics Analysis

For Morris water maze analysis, escape latency was analyzed by repeated measures two-way ANOVA plus Bonferroni multiple comparisons, with treatment as between subjects factor and testing day as within subjects factor. Data for probe trail were analyzed by one-way ANOVA plus Bonferroni multiple comparisons. For gene array and qPCR, data were analyzed using one-way ANOVA with an ad hoc comparison using Turkey’s analysis. All the statistical analysis was performed by using GraphPad Prism (version 6, GraphPad Software, La Jolla, CA, USA). Difference was considered to be significant at *p* < 0.05.

## 3. Results

### 3.1. General Toxicity

As shown in Table 1, none of DEHP doses affected the birth rates (number of dams that delivered litters divided by the number of dams with an established pregnancy as defined by the presence of a vaginal plug) and the number of pups per dam. Pups in the three groups were all survived. 10 mg/kg DEHP did not affect the birth weights of pups. In 750 mg/kg DEHP group, the average birth weight was significantly decreased to 81.5% of that of control group. It suggests that 750 mg/kg DEHP causes low birth weight of pups.

**Table 1:** General toxicity of DEHP.

Parameters	Dose of DEHP (mg/kg)		
	0	10	750
Number of dams	14	14	14
Birth rate (%)	100	100	100
Number of pups per dam	14.0 ± 0.2	13.1 ± 0.2	12.5 ± 0.3
Pup’s birth weight (g)	6.81 ± 0.04	6.89 ± 0.03	5.55 ± 0.05 ***

Mean ± SEM, *** *p* < 0.001 when compared to with control group (DEHP 0 mg/kg).

### 3.2. Performance in Spatial Reference Memory

**Figure 1:**
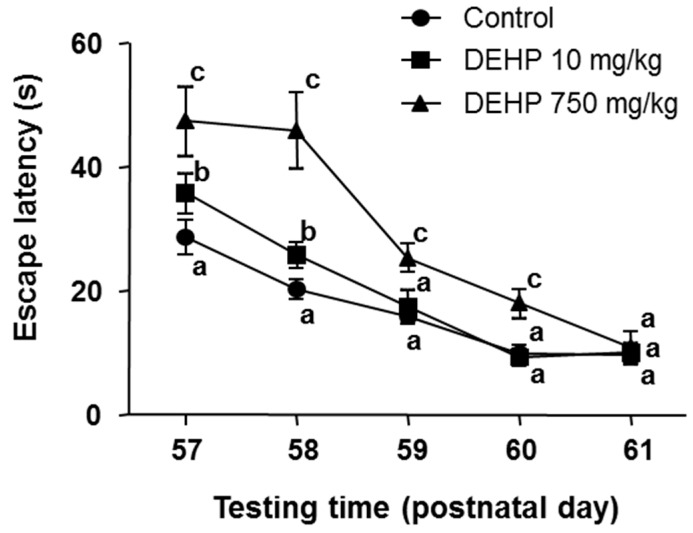
Mean escape latency to platform in each consecutive day. Mean ± SEM, *n* = 10. Identical letters at each time point designate no significant difference between two groups at *p* < 0.05.

The repeated measures using two-way ANOVA for escape latency indicate that the between subjects factor of DEHP treatment was significant (*F* (2, 27) =255.9, *p* < 0.0001), as was the within subjects factor of testing day (*F* (4, 108) = 476.1, *p* < 0.0001). The interaction between these two factors was also significant (*F* (8, 108) = 29.21, *p* < 0.0001). Bonferroni multiple comparisons were used for further analysis (compare each group to other two groups). When compared to control, the latencies in 10 mg/kg DEHP group were significantly prolonged on PND 57 (*p* < 0.0001) and PND 58 (*p* < 0.001), and in 750 mg/kg DEHP group those were significantly prolonged from PND 57 through PND 60 (*p* < 0.0001). When compared to 10 mg/kg DEHP group, the latencies in 750 mg/kg DEHP group was also prolonged from PND 57 through PND 60 (*p* < 0.0001). No significant differences were detected among these three groups on PND 61 (Figure 1). There is no significant difference in search length between control and DEHP-treated offspring (29.23 ± 0.83 (mean ± SEM), 29.29 ± 0.83, and 28.38 ± 0.90 m for control, 10 and 750 mf/kg DEHP, respectively), indicating that there is non-hyperkinesis after DEHP treatment.

### 3.3. Performance in Probe Trials

One-way ANOVA with Bonferroni test (comparison of each group to other two groups) was used for analyzing the times across the platform and search time in quadrant I. Both 10 mg/kg DEHP group (*p* < 0.05) and 750 mg/kg DEHP group (*p* < 0.01) had much fewer times across the platform, when compared to control. 

**Figure 2:**
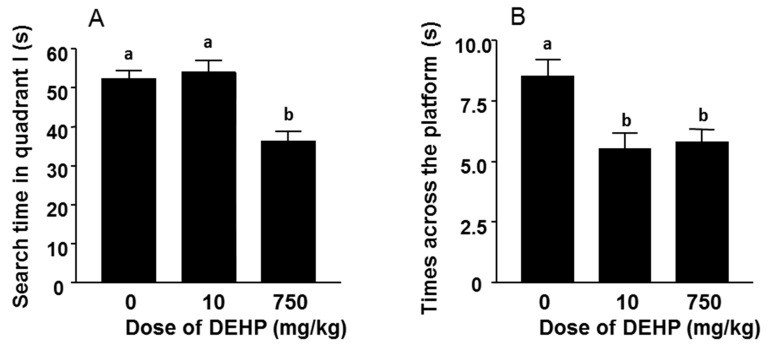
Search time in quadrant I and times across the platform. Mean ± SEM, *n* = 10. Panel A, Search time in quadrant I; Panel B, Times across the platform. Identical letters designate no significant difference between two groups at *p* < 0.05.

No significant difference was observed between low dose (10 mg/kg DEHP) group and high dose (750 mg/kg DEHP) group in times across the platform. 750 mg/kg DEHP group had much less search time (70% of that of control) in quadrant I when compared to control (*p* < 0.001) or 10 mg/kg DEHP group (*p* < 0.001). No significant difference was observed between 10 mg/kg DEHP group and control group in search time in quadrant I (Figure 2).

### 3.4. Effects of in Utero DEHP Exposure on Gene Expression Profiling of Neonatal Brain 

**Figure 3:**
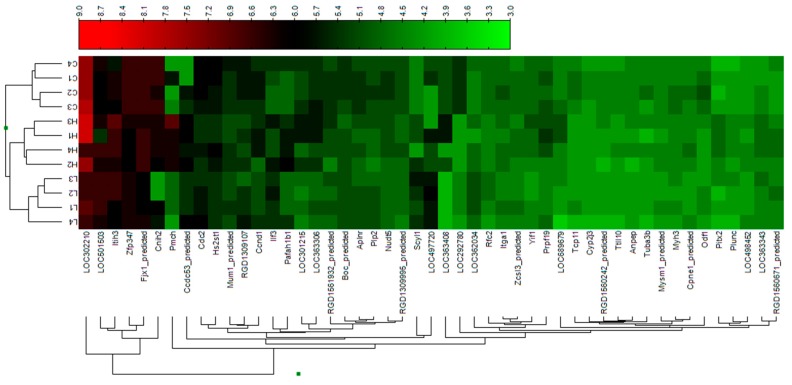
Visualization of a set of altered genes by hierarchical clustering analysis. Samples are depicted in the columns. The log intensities of a set of genes which were statistically changed ≥1.5 fold were hierarchically clustered by computing the distance between two points if a grid-like path is followed and average linkage. Each column represents individual animal. C, L and H represent 0 (control), 10 and 750 mg/kg DEHP, respectively.

Illumina RatRef-12 expression BeadChip was used to determine the effects of DEHP on the expression levels of genes in male neonatal brain. A genome-wide expression containing 21,910 gene probes was analyzed. 10,034 genes were detected according to the detection *p*-value (see Materials and Methods). We further compared DEHP-treated groups to control. After 10 mg/kg DEHP treatment, six genes were significantly up-regulated and 29 genes down-regulated. After 750 mg/kg DEHP treatment, nine genes were significantly up-regulated and 16 genes down-regulated. These genes with ≥1.5 fold changes are listed in Table 2 (up-regulated) and Table 3 (down-regulated). In these changed genes, two genes (*Cdc2*, *Ccnd1*) related to the cell cycle were down-regulated in 10 or 750 mg/kg DEHP group. Heat map was generated for visualization of a set of statistically changed genes of different samples by hierarchical clustering analysis (Figure 3). The log intensities of a set of genes which were statistically changed ≥1.5 fold were hierarchically clustered by computing the distance between two points if a grid-like path is followed and average linkage. According to the sample clustering analysis, the gene expression patterns were different in these three groups. Several pathways, including cell cycle, metabolism and transcription process, were involved (Table 2 and Table 3).

**Figure 4:**
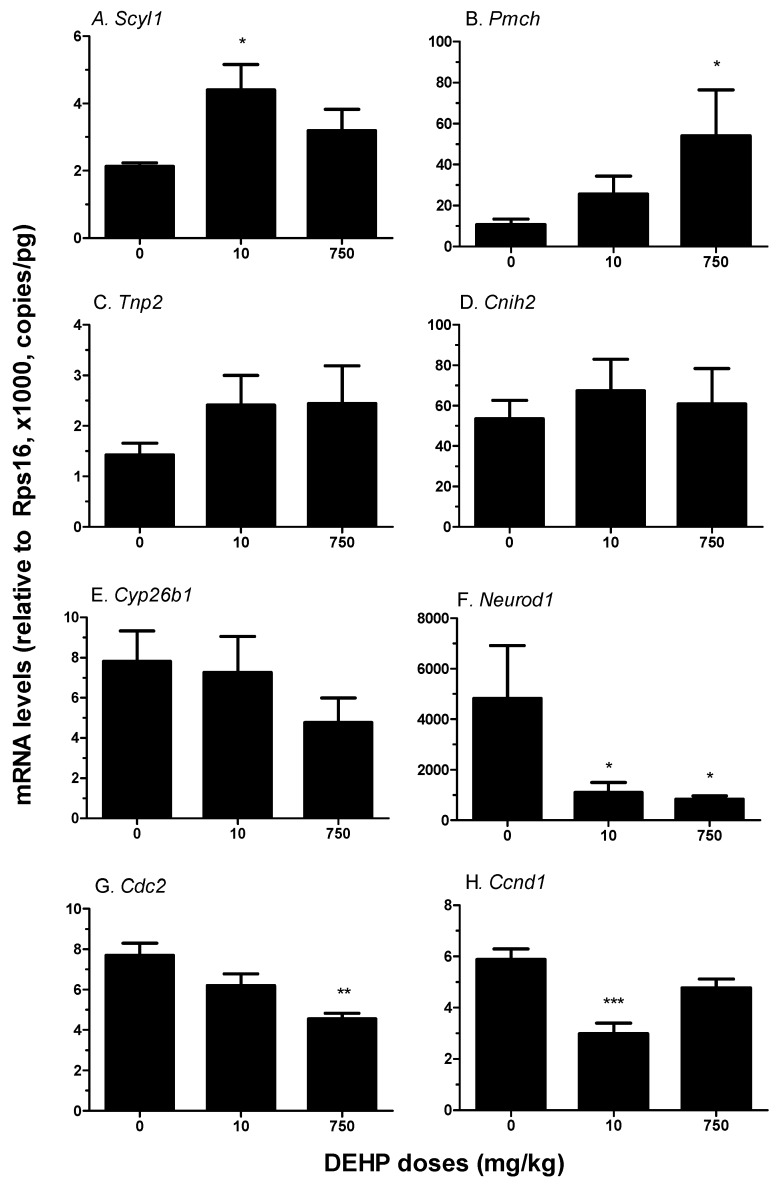
qPCR validation of gene expression. Panel **A–H** shows the gene names (Scy1 = SCY1-like 1; Pmch = Pro-melanin-concentrating hormone; Tnp2 = Transition protein 2; Cornichon homolog 2; Cyp26b1 = Cytochrome P450 26B1; Neurod1 = Neurogenic differentiation 1; Cdc2 = Cyclin dependent kinase 1; Ccnd1 = Cyclin D1). Mean ± SEM, *n* = 6. *, **, and *** designate significant difference compared to control at *p* < 0.05, 0.01, 0.001, respectively.

**Table 2:** Up-regulated genes in rat brain after DEHP exposure *in utero*.

Gene		Dose of DEHP (mg/kg)		
Symbol	Name	0	10	750
**Membrane ligand/receptor/membrane-mediated signaling**				
Pafah1b1	Platelet-activating factor acetylhydrolase IB subunit alpha	132 ± 31	134 ± 26	283 ± 28 (+2.1) *
Pmch	Pro-melanin-concentrating hormone	120 ± 68	79 ± 11	692 ± 98 (+5.8) **
**Transcription factor/RNA processing protein**				
Scyl1	SCY1-like 1	63 ± 3	208 ± 24 (+3.3) ***	135 ± 36 (+2.1) **
Pitx2	Pituitary homeobox 2	31 ± 7	32 ± 7	81 ± 11 (+2.6) ***
**Protein transporters**				
Ccdc53_predicted	Coiled-coil domain containing 53	146 ± 61	262 ± 51 *	399 ± 35 (+2.7) ***
**Unknown function**				
LOC497720	Unknown	118 ± 44	319 ± 36 (+2.7) ***	175 ± 62

* *p* < 0.05, ** *p* < 0.01, *** *p* < 0.001 when compared to control group (DEHP 0 mg/kg); () Fold change.

**Table 3:** Down-regulated genes in rat brain after DEHP exposure *in utero*.

Gene		Dose of DEHP (mg/kg)		
Symbol	Name	0	10	750
**Membrane ligand/receptor/membrane-mediated signaling**				
Cnih2	Cornichon homolog 2	715 ± 54	212 ± 103 (−3.4) ***	557 ± 60
**Metabolic pathway**				
Cyp2j3	P450, family 2j, polypeptide 3	56 ± 5	30 ± 6 (−2.0) **	39 ± 1
Hs2st1	Heparan sulfate 2-O-sulfotransferase 1	382 ± 32	202 ± 6 (−2.0) **	275 ± 46 *
**Cellular structure protein**				
Odf1	Outer dense fiber protein 1	110 ± 14	56 ± 9 (−2.0) **	39 ±3 (−2.8) **
Tuba3b	Tubulin, alpha 3B	64 ± 9	31 ± 1 (−2.1) **	42 ± 9
**Cell cycle regulators**				
Neurod1	Neurogenic differentiation 1	429 ± 98	307 ± 88 (−1.4)	214 ± 32 (−2.0) **
Ccnd1	Cyclin D1	269 ± 12	144 ± 4 (−1.9) **	218 ± 6 (−1.3)
Cdc2	Cyclin dependent kinase 1	398 ± 15	301 ± 8 (−1.3)	214 ± (−1.9) **
**Protein transporters**				
Tcp11	T-complex protein 11 homolog	83 ± 14	41 ± 4 (−2.0) **	33 ± 1 (−2.5) ***
Yif1	Yip1 interacting factor homolog	119 ± 5	53 ± 10 (−2.3) **	107 ± 13
**Unknown function**				
LOC292780	Unknown	219 ± 11	50 ± 4 (−4.4) ***	43 ± 2 (−5.1) ***
LOC301215	Unknown	195 ± 21	85 ± 6 (−2.3) ***	243 ± 66
LOC302210	Unknown	2341 ± 186	917 ± 26 (−2.6) **	2682 ± 674
LOC363306	Unknown	224 ± 15	118 ± 9 (−2.0) *	261 ± 36
LOC363408	Unknown	148 ± 7	19 ± 2 (−7.8) ***	190 ± 60
LOC689679	Unknown	79 ± 4	33 ±8 (−2.4 ) ***	105 ± 26

* *p* < 0.05, ** *p* < 0.01, *** *p* < 0.001 when compared to control group (DEHP 0 mg/kg); () Fold change.

Eight genes were selected for validation by qPCR (Figure 4). QPCR results for *Pmch* and *Scyl1* was coincident with that of the gene array, with *Pmch* up-regulated in 750 mg/kg group (*p* < 0.05) and *Scyl* up-regulated in 10 mg/kg group (*p* < 0.05). Expressions of *Cyp26b1* and *Tnp2* were not significantly changed in any DEHP groups, which is also consistent with that of gene array. *Neurod1* was significantly downregulated in both 10 and 750 mg/kg. *Ccnd1* was downregulated in 10 mg/kg DEHP and *Cdc2* was downregulated in 750 mg/kg DEHP, which were in parallel with microarray data. qPCR didn’t detect any difference in *Cnih2* expression.

## 4. Discussion

The present study provided evidence that *in utero* exposure to DEHP resulted in spatial memory and learning ability deficits of male offspring at adulthood. Gene expression profiling for brain genes of neonatal male pups showed that DEHP exposure altered gene expression profiles of neonatal rat brain.

The Morris water maze was used for the evaluation of learning and spatial memory abilities of male offspring at adulthood after 10 days of DEHP exposure *in utero*. Both doses of DEHP showed significant deficits in cognitive function when compared with the control group, and this impairment showed dose-dependent with longer escape latency, shorter search time in quadrant I in 750 mg/kg DEHP. A recent study in mice also showed that in the Morris water maze test, *in utero* and lactational exposure to 50 and 200 mg/kg/d DEHP significantly prolonged the time of searching for the hidden platform and reduced the time staying in the target quadrant during a probe trial of male mice, but not female mice [19]. However, in another study in mice, *in utero* and lactational exposure to 30 mg/kg/d DEHP did not alter spatial learning and memory in offspring [20]. Epidemiological studies also showed that DEHP exposure impaired cognitive functions, such as executive functioning [21], attention defect [22] and declined intelligence [23] in children. Several studies using animal models have demonstrated that the fetal exposure to phthalates is associated with later-onset diseases, including the reduced serum testosterone and infertility [24,25].

We further hypothesized that the altered cognitive function at adulthood after *in utero* DEHP exposure may be caused by the impairment of fetal brain development. It was true that acute exposure to DEHP (10 mg/kg) prepubertally reduced axonal markers in the CA3 distal *stratum oriens* and reduced cell density of both immature and mature neurons in the *dentate gyrus* and CA3 as well the expression level of brain-derived neurotrophic factor in male not female rats [26,27]. To explore this, we performed genome-wide gene expression analysis of the neonatal brain after *in utero* DEHP exposure. Indeed, several pathways, including cell cycle, metabolism and transcription processing, were significantly altered in neonatal brain even at low dose of DEHP (Table 2 and Table 3). During fetal life, neurogenesis is very important for populating the growing brain with neurons via proliferation and differentiation of neural stem and progenitor cells [28]. Neurogenic differentiation 1 (*Neurod1*) is a member of the NeuroD family of basic helix-loop-helix (bHLH) transcription factors. It has been shown that NEUROD1 interacted with cyclin D1 for cell differentiation [29]. Cell division control protein 2 homolog (*Cdc2*) and cyclin D1 (*Ccnd1*), two important genes for stem and progenitor cell proliferation, were significantly down-regulated after DEHP exposure. Cyclin D1, a member of cyclins, acts as a regulator of CDK kinases by conjugating with CDKs [30]. Cyclin D1 binds to CDK (including CDC2) to form a complex to regulate cell cycle G1/S transition and thus they are important for neural proliferation [31]. Thus the down-regulation of either of them can significantly reduce cell proliferation. It is true that *in vitro* DEHP treatment significantly inhibited proliferation of the PC12 cell, a neuron-related cell line [32]. Many growth factors induce the expression of *Ccnd1*, including epidermal growth factor or heparin-binding epidermal growth factor-like growth factor (HB-EGF) [33]. Cornichon-like protein (*Cnih2*) has been shown to facilitate secretion of HB-EGF, thus regulating proper development of cranial nerves [34]. We found that *Cnih2* expression was down-regulated 3.4 fold after 10 mg/kg DEHP exposure. This down-regulation is concurs with the significant down-regulation of *Ccnd1* in the 10 mg/kg DEHP group. Interestingly, the expressions of both *Ccnd1* and *Cnih2* were not significantly altered in the 750 mg/kg DEHP group. The underlying mechanism for this discrepancy is unclear. However, generally speaking, more genes were down-regulated in 10 mg/kg DEHP group (29 genes) *vs.* 750 mg/kg (16 genes), suggesting that low dose of DEHP causes more broad alteration of gene expressions.

Some gene expressions were also up-regulated after DEHP treatment. One of them is *Pmch*, which is majorly expressed within the lateral hypothalamus. Its product, melanin-concentrating hormone (MCH), involved in the regulation of feeding behavior, mood and metabolism [35]. In the present study, *Pmch* was up-regulated about 6-fold in the 750 mg/kg DEHP group, indicating that high-dose DEHP exposure may also regulate the feeding behavior, mood and metabolism. In addition, there were some other critical gene expressions up-regulated in DEHP groups, including some transcriptional factors, like *Pitx2*, *Scyl1* and *Ilf3*.

The changed gene expression patterns partially but not fully explain the deficits of cognitive function in later life after *in utero* DEHP exposure. This may be caused by significant alterations of metabolism and the endocrine system after exposure to high doses of DEHP. For example, higher dose of DEHP caused more severe reduction of androgen biosynthesis in the testes. The studies from our lab and others have demonstrated that high dose DEHP exposure (234–750 mg/kg) *in utero* resulted in low testosterone production in the fetal testes [1,36,37]. Androgens are neuroprotectants or neuromodulators, and play a pivotal role during the “organizational/developmental” phase. Androgen receptor has been shown to modulate GABAergic system in central nervous system (see review by Genazzani *et al.* [38]). In this regard, males generally perform better than females in spatial learning tests [39,40]. Phthalate-mediated deficits in cognitive deficits in the male offspring might be indirectly linked to its actions on endocrine system. A recent epidemiology study suggested that prenatal exposure to phthalates was associated with less male-typical play behavior in boys [13].

## 5. Conclusions

Both 10 and 750 mg/kg DEHP exposures *in utero* changed gene expression patterns. Some critical genes, like *Ccnd1* and *Cdc2* for neuron proliferation, were significantly down-regulated. Fetal exposure to DEHP might result in learning and spatial memory deficits observed at adulthood.
